# Supplementary material for: A Pan-Amazonian species delimitation: high species diversity within the genus Amazophrynella (Anura: Bufonidae)
Source: PeerJ. 2018 Jul 9;6:e4941. doi: 10.7717/peerj.4941 (PMC6042491; doi:10.7717/peerj.4941)
Supplement: Supplementary file 1 [file peerj-06-4941-s001.odt]

SUPPORTING INFORMATION

A Pan-Amazonian species delimitation: high species diversity within the genus Amazophrynella (Anura: Bufonidae)

APPENDIX S1 – Exanimated specimens 

A. minuta.—BRAZIL: Amazonas State: Uauapés River, Taracuá, INPA–H 32720–23, INPA–H 32725–26, INPA–H 32728–30, INPA–H 32732, INPA–H 32733, INPA–H 32735–40, NHMG 462, NHMG 463, NHMG 464.; Sao Gabriel da Cachoeira, INPA–H32731, INPA–H 32724, INPA–H 32734, Japura River, INPA–H 355507–12, INPA–H 35494–98. 

A. bokermanni.—BRAZIL: Pará State, Juriti (near 30 km from type locality) INPA–H 31861–65, Amazonas State, Autazes: INPA–35529–30; Pará State: Xingu River INPA 35473–77.

A. vote.—BRAZIL: Mato Grosso State: Cotriguaçu, Fazenda São Nicolau, UFMT–A 11138 (Holotype); UFMT 11136, UFMT 11142, UFMT 11145–50, UFMT 11152–55, UFMT 4412; Manicoré, Madeira River, INPA–H 12255–56, 12331, 12342–43, 12366–67 (Paratypes); Parque Estadual do Guariba INPA–H 21558, Novo Aripuanã, Aripuanã River, INPA–H 12326 (Paratype) INPA–H 35540−50; Estado Amazonas: Tapauá, Parque Nacional Nascentes do Lago Jari, INPA–H 27412, 27417−19, 27421−23, 27425−26 (Paratypes), Tapauá, Purus river, INPA–H 35551−53, Manaquiri, INPA–H 35535−39; Estado do Amazonas: Matupiri, INPA–H 31867, INPA–H 31868, INPA–H 31870–75, INPA–H 31877–80, INPA–H 31882, INPA–H 31883–66.

A. manaos.—BRAZIL: Amazonas State: Campus da Universidade Federal do Amazonas, INPA–H 31866 (Holotype), INPA–H 6983–64, INPA–H 6987, INPA–H 7797 (paratypes); Presidente Figueiredo, INPA–H 20986, INPA–H 21217, INPA–H 29568–72, INPA–H 30575–77, INPA–H 30572–73 (Paratypes); Reserva Florestal Adolpho Ducke, INPA–H 21028, INPA–H 21170, INPA–H 21060, INPA–H 31866, INPA–H 21007–13, INPA–H 20963–INPA-H 20990.

A. matses.—PERU: Department Loreto: Requena, Nuevo Salvador, MZUNAP 921(Holotype), MZUNAP 922–23, MZUNAP 925–27, MZUNAP 934, MZUNAP 936, MZUNAP 938, MZUNAP 940, MZUNAP 943–44, MZUNAP 948, MZUNAP 952–53, MZUNAP 955, MZUNAP 958 (paratopotypes); Jenaro Herrera MZUNAP 928–31, MZUNAP 933, MZUNAP 935, MZUNAP 937, MZUNAP 939, MZUNAP 950, MZUNAP 941–42, MZUNAP 946–47, MZUNAP 949 (Paratypes).

A. amazonicola.—PERU: Department Loreto: San Juan Bautista, Puerto Almendra MZUNAP 901 (Holotype), MZUNAP 906–07, MZUNAP 910–11, MZUNAP 913–17; MZUNAP 110; MZUNAP 889 (paratopotypes); 58 km of Iquitos–Nauta highway on Fundo Zamora, MZUNAP 887–88, MZUNAP 900, MZUNAP 902, MZUNAP 886, MZUNAP 905, MZUNAP 908, MZUNAP 919–20, MZUNAP 924 (Paratypes); Maynas, Nauta, MZUNAP 909, MZUNAP 918; Fundo UNAP, MZUNAP 242 (Paratype).

Amazophrynella javierbustamantei.—PERU: Department Madre de Dios: Tambopata, Quebrada Guacamayo, MHNC 8331(Holotype), MHNC 8316, MHNC 8238, MHNC 8362–63, MHNC 8245, MHNC 8484, La Pampa, MHNC 1101–04; Nuevo Arequipa, MHNC 8245, MHNC 8331, MHNC 8238, MHNC 8354, MHNC 8484, Rio Tambopata, MHNSM 9633, MHNSM 9635, MHNSM 9640–42, MHNSM 9644, MHNSM 9646–48; Manu, Inambari, MHNSM 17993; La Convencion, Camana, MHNSM 2565; Mapi, MHNC 9939–40. Departamento: Junin: Tambo Poyeni, MHNC 9387; Tsoroja, MHNC 9626, MHNC 9754, MHNC 9756–57, MHNC 9679, MHNC 9680. Department: Cusco: Urubamba, Urubamba River, MHNC 9626, MHNC 9686–87.

Amazophrynella moisesii.—BRAZIL: Acre: Parque Nacional da Serra do Divisor: Igarapé Ramon: UFAC-H 2815 (Holotype), UFAC-H 1375, UFAC-H 2772–2773, UFAC-H 2603, UFAC-H 2607, UFAC-H 3573–UFAC-H 3575, UFAC-H 2690, UFAC-H 2692, UFAC-H 2815–2817, UFAC-H 1698; Igarapé Anil: UFAC-H 1337–1343; Zé Luiz lake: UFAC-H 1774–1775; Môa river: UFAC-H 1493, UFAC-H 2687–2697. Reserva Extrativista Alto do Juruá: UFAC-H 822–823, UFAC-H 878–879, UFAC-H 2606–2611 Gregório Forest Reverse: UFAC-H 5678.

Amazophrynella teko.—BRAZIL: Amazonas state, Trombetas river: INPA-H 35513–35530, Amapa state: UFPA AA 604. French Guiana: District of Camopi: Alikéné: MNHN 2015.136 (Holotype). District of Saint Laurent du Maroni: Mitaraka layon: MNHN 2015.137, MNHN 2015.138, MNHN 2015.139, MNHN 2015.140, MNHN 2015.141, MNHN 2015.142, MNHN 2015.143, Pic Coudreau du Sud: MNHN 2015.152, MNHN 2015, Flat de la Waki: INPA–H 36598. District of Camopi: Mitan: INPA–H 36596, MNHN 2015.144, MNHN 2015.145, MNHN 2015.146, MNHN 2015.147, MNHN 2015.148, MNHN 2015.149, MNHN 2015.150. District of Saint Georges: Saint Georges: MNHN 2015.151, Mémora: MNHN 2015.154, MNHN 2015.155, Saut Maripa: INPA–H 36597, INPA–H 36610, INPA–H 36599, INPA–H 36601, INPA–H 36600.

Amazophrynella xinguensis.—BRAZIL: Para state: Sustainable Development Project (PDS) Virola Jatobá: INPA–H 35471 (Holotype), INPA–H 35484, INPA–H 35485 INPA–H 35473, INPA–H 35474, INPA–H 35475, INPA–H 35476, INPA–H 35477, INPA–H 35478, INPA–H 35479, INPA–H 354780, INPA–H 35481, INPA–H 35483, INPA–H 35490, INPA–H 35491, INPA–H 3592, Fazenda Paraiso: INPA–H 35493, INPA–H 35472, Ramal dos Cocos: INPA–H 35486, INPA–H 35487, INPA–H 3588, INPA–H 35489. 

Amazophrynella siona.—ECUADOR: Province of Orellana: Yasuni National Park: QCAZ 27790 (Holotype), QCAZ 11981, QCAZ 51068, QCAZ 21425, QCAZ 21431, QCAZ 11973, QCAZ 11979. Provincia Sucumbios: Reserva de Producción Faunística Cuyabeno: QCAZ 52433–34, QCAZ 37758–59, QCAZ 37761, QCAZ 6071, QCAZ 6091, QCAZ 6095, QCAZ 6097, QCAZ 6105, QCAZ 6111, QCAZ 6113, QCAZ 6118, QCAZ 6127, QCAZ 6128, Santa Cecilia, QCAZ 4469, QCAZ 4472, Tarapoa: QCAZ 36331, QCAZ 36336, QCAZ 36338, QCAZ 36357. Provincia de Pastaza: Community of Kurintza: QCAZ  56342, QCAZ 56354, QCAZ 56361, Villano community, AGIP oil company: QCAZ 38599, QCAZ 38679, QCAZ 38722, Around Villano community, AGIP oil company: QCAZ 38642, Community of Kurintza: QCAZ 38809, QCAZ 54213, Bataburo Lodge: QCAZ 39408, Lorocachi: QCAZ 8902, Lorocachi: QCAZ 56165, Canelos: QCAZ 52819, QCAZ 52823, QCAZ 17391. Provincia Orellana: Tambococha: QCAZ 55345, Garzacocha: QCAZ 20504, Yuriti: QCAZ 10526, Kapawi Lodge: QCAZ 8725, QCAZ 25504, QCAZ 25533 10 km from Puyo: QCAZ 7135. Provincia Morona Santiago: Pankints: QCAZ 46430. PERU: Department Loreto: Teniente Lopez: MHNC 7611, MHNC 7685, MHNC 7686, MHNC 7698, MHNC 7699, MHNC 7700, Jibarito: MHNC 7786, MHNC 7809, MHNC 7814. Shiviyacu: MHNC 14730, near Corrientes River: MHNC 6292. 

Amazophrynella moisesii.—BRAZIL: Acre state: Reserva Extrativista Alto do Juruá: UFAC-RB 823, UFAC-RB 878–879, Parque Nacional da Serra do Divisor: Igarapé Anil: UFAC-RB 1337–1341, UFAC-RB 1343, Zé Luiz lake: UFAC-RB 1774–1775, Igarapé Ramon: UFAC-RB 1375, UFAC-RB 2772–2773, UFAC-RB 2816–2817, Môa river: UFAC-RB 1493, UFAC-RB 2687–2697, Gregório forest Reserve: UFAC-RB 5678
