# Supplementary material for: A Pan-Amazonian species delimitation: high species diversity within the genus Amazophrynella (Anura: Bufonidae)
Source: PeerJ. 2018 Jul 9;6:e4941. doi: 10.7717/peerj.4941 (PMC6042491; doi:10.7717/peerj.4941)
Supplement: Supplementary file 2 [file peerj-06-4941-s002.odt]

SUPPORTING INFORMATION

A Pan-Amazonian species delimitation: high species diversity within the genus Amazophrynella (Anura: Bufonidae)

APPENDIX 2 – PCR AMPLIFICATION CONDITION AND Sampling and source of DNA

PCR amplification condition 

Total DNA was extracted from muscle tissue using standard phenol/chloroform extraction (Sambrook et al., 1989).. Amplification of three genes (12S, 16S and COI) from this study was carried out under the following conditions: 60 s hot start at 92°C followed by 35 cycles of 92°C (60 sec), 50°C (50 sec) and 72°C (1.5 min). Final volume of the PCR reaction for all genes was 12 μl and contained 4.4 μL of ddH2O, 1.5 μL of 25 mM MgCl2, 1.25 μL of 10 mM dNTPs (2.5mM each dNTP), 1.25 μL of 10x buffer (75 mM Tris HCl, 50 mM KCl, 20 mM (NH4)2SO4), 1 μL of each 2 μM primer, 0.3 μL of 5 U/μL DNA Taq Polymerase (Fermentas, Lithuania) and 1 μL of DNA (about 30 ng/μL). Sequencing reactions were carried out according to the manufacturer's recommendation for the ABI BigDye Terminator cycle sequencing mix, using amplification primers and annealing temperature of 50°C. Sequencing reactions were precipitated using standard EDTA/EtOH protocol, and resolved in an ABI 3500 automatic sequencer. 

Supplementary Table S2a.  List of genetic sample of Amazophrynella taxa used in this study, with localities, country, geographic coordinates, museum voucher, taxonomic status, discrete area (see Materials and Methods). 

.

| Sample | Locality, Country | lat | long | 16S | 12S | COI | Voucher number | Status | Area |
| --- | --- | --- | --- | --- | --- | --- | --- | --- | --- |
| A. sp1. | Trombetas River, Pará, Brazil | -1.197472 | -56.670022 | MH270091 | MH269906 | MH270307 | INPA-H 35513 |  | NE |
| A. sp1. | Trombetas River, Pará, Brazil | -1.197472 | -56.670022 | MH270092 | MH269907 | MH270308 | INPA-H 35514 |  | NE |
| A. sp1. | Trombetas River, Pará, Brazil | -1.197472 | -56.670022 | MH270093 | MH269908 | MH270309 | INPA-H 35515 |  | NE |
| A. sp1. | Trombetas River, Pará, Brazil | -1.197472 | -56.670022 | MH270094 | MH269909 | MH270310 | INPA-H 35516 |  | NE |
| A. sp1. | Trombetas River, Pará, Brazil | -1.197472 | -56.670022 | MH270095 | MH269910 | MH270311 | INPA-H 35517 |  | NE |
| A. sp1. | Trombetas River, Pará, Brazil | -1.197472 | -56.670022 | MH270096 | MH269911 | MH270312 | INPA-H 35518 |  | NE |
| A. sp1. | Trombetas River, Pará, Brazil | -1.197472 | -56.670022 | MH270097 | MH269912 | MH270313 | INPA-H 35519 |  | NE |
| A. sp1. | Trombetas River, Pará, Brazil | -1.197472 | -56.670022 | MH270098 | MH269913 | MH270314 | INPA-H 35520 |  | NE |
| A. sp1. | Trombetas River, Pará, Brazil | -1.197472 | -56.670022 | MH270099 | MH269914 | MH270315 | INPA-H 35521 |  | NE |
| A. sp1. | Trombetas River, Pará, Brazil | -1.197472 | -56.670022 | MH270100 | MH269915 | MH270316 | INPA-H 35522 |  | NE |
| A. sp1. | Trombetas River, Pará, Brazil | -1.430833 | -56.747278 | MH270101 | MH269916 | MH270317 | INPA-H 35523 |  | NE |
| A. sp1. | Trombetas River, Pará, Brazil | -1.430833 | -56.747278 | MH270102 | MH269917 | MH270318 | INPA-H 35524 |  | NE |
| A. sp1. | Trombetas River, Pará, Brazil | -1.430833 | -56.747278 | MH270103 | MH269918 | MH270319 | INPA-H 35525 |  | NE |
| A. sp1. | Trombetas River, Pará, Brazil | -1.430833 | -56.747278 | MH270104 | MH269919 | MH270320 | INPA-H 35526 |  | NE |
| A. sp1. | Trombetas River, Pará, Brazil | -1.430833 | -56.747278 | MH270105 | MH269920 | MH270321 | INPA-H 35527 |  | NE |
| A. sp1. | Trombetas River, Pará, Brazil | -1.430833 | -56.747278 | MH270106 | MH269921 | MH270322 | INPA-H 35528 |  | NE |
| A. sp1. | Trombetas River, Pará, Brazil | -1.430833 | -56.747278 |  | MH269925 |  | INPA-H 35529 |  | NE |
| A. teko sp. nov. | Mitaraka, French Guyana | 2.235833 | -54.449167 | EU201057 |  | JN867544 | AF3035 |  | NE |
| A. teko sp. nov. | Oiapoque, Brazil | 3.824357 | - 53.835377 | KR811165 |  |  | MTR24057 |  | NE |
| A. teko sp. nov. | Lourenco, Brazil |  |  | KR811166 |  |  | MTR13918 |  | NE |
| A. teko sp. nov. | Savane Roche Virgine, French Guyana | 4.134751 | - 52.086610 | KR811167 |  |  | PG776 |  | NE |
| A. teko sp. nov. | Saut Maripa, French Guyana | 3.806111 | -51.893333 | KR811168 |  |  | AF0761 |  | NE |
| A. teko sp. nov. | Oiapoque, Brazil | 3.824357 | - 53.835377 | KR811165 |  |  | MTR24242 |  | NE |
| A. teko sp. nov. | Savane Roche Virgine, French Guyana | 4.134751 | - 52.086610 | JN867570 |  | JN867543 | 296MC |  | NE |
| A. teko sp. nov. | Pic Courdeau do Sud, French Guyana | 2.253889 | -54.351111 | MH270107 | MH269922 | MH270323 | MNHN 2015.152 |  | NE |
| A. teko sp. nov. | Flat de la Waki, French Guyana | 3.087500 | -53.403333 | MH270108 | MH269923 | MH270324 | INPA– H 36598 |  | NE |
| A. teko sp. nov. | Lac Toponowini, French Guyana | 3.087500 | -53.403333 | MH270109 | MH269924 | MH270325 | AF0900 |  | NE |
| A. teko sp. nov. | Amapá, Brazil | 0.231818 | -51.863490 | MH270110 | MH269905 | MH270306 | UFPA AA 604 |  | NE |
| A. manaos | Campus UFAM, Amazonas, Brazil | -3.098889 | -59.969167 | KF433956 | MH269887 | MH270288 | INPA-H 6987 | Paratopotype | NE |
| A. manaos | Fazenda UFAM, Amazonas, Brazil | -2.588889 | -60.115278 | KF433958 | MH269887 | MH270289 | INPA H 7798 |  | NE |
| A. manaos | Fazenda UFAM, Amazonas, Brazil | -2.588889 | -60.115278 | KF433959 | MH269888 | MH270290 | INPA-H 30577 | Paratype | NE |
| A. manaos | Presidente Figueiredo, Amazonas, Brazil | -1.938889 | -60.037222 | KF433961 | MH269889 | MH270291 | INPA-H 30573 | Paratype | NE |
| A. manaos | Mineração Taboca, Amazonas, Brazil | -0.716389 | -60.173333 | KF433963 | MH269890 | MH270292 | INPA-H 30575 | Paratype | NE |
| A. manaos | Rio Cuieiras, Amazonas, Brazil | -2.724167 | -60.406111 | KF433964 | MH269891 | MH270293 | INPA-H 1859 | Paratype | NE |
| A. manaos | Campus UFAM, Amazonas, Brazil | -3.098889 | -59.969167 | KF433954 | MH269892 | MH270294 | INPA-H 6983 |  | NE |
| A. manaos | Campus UFAM, Amazonas, Brazil | -3.098889 | -59.969167 | KF433955 | MH269893 | MH270295 | INPA-H 6984 |  | NE |
| A. manaos | Presidente Figueiredo, Amazonas, Brazil | -1.938889 | -60.037222 | KF433968 | MH269894 | MH270296 | INPA-H 29570 | Paratype | NE |
| A. manaos | Presidente Figueiredo, Amazonas, Brazil | -1.938889 | -60.037222 | MH270082 | MH269895 | MH270297 | INPA–H 20986 | Paratype | NE |
| A. manaos | Presidente Figueiredo, Amazonas, Brazil | -1.938889 | -60.037222 | MH270083 | MH269896 | MH270298 | INPA–H 21217 | Paratype | NE |
| A. manaos | Presidente Figueiredo, Amazonas, Brazil | -1.938889 | -60.037222 | MH270084 | MH269897 | MH270299 | INPA–H 29568 | Paratype | NE |
| A. manaos | Presidente Figueiredo, Amazonas, Brazil | -1.938889 | -60.037222 | MH270085 | MH269898 | MH270300 | INPA–H 29569 | Paratype | NE |
| A. manaos | Presidente Figueiredo, Amazonas, Brazil | -1.938889 | -60.037222 | MH270086 | MH269899 | MH270301 | INPA–H 29570 | Paratype | NE |
| A. manaos | Presidente Figueiredo, Amazonas, Brazil | -1.938889 | -60.037222 | MH270087 | MH269900 | MH270302 | INPA–H 29571 | Paratype | NE |
| A. manaos | Presidente Figueiredo, Amazonas, Brazil | -1.938889 | -60.037222 | MH270088 | MH269901 | MH270303 | INPA–H 29572 | Paratype | NE |
| A. manaos | Presidente Figueiredo, Amazonas, Brazil | -1.938889 | -60.037222 | MH270089 | MH269902 | MH270304 | INPA–H 30575 | Paratype | NE |
| A. manaos | Presidente Figueiredo, Amazonas, Brazil | -1.938889 | -60.037222 | MH270090 | MH269903 | MH270305 | INPA–H 30576 | Paratype | NE |
| A. aff. vote sp.1 | Parque Nacional Nascentes do Lago Jari, Brazil | -4.832444 | -61.002528 | MH270029 | MH269839 | MH270241 | INPA-H 27412 | Paratype | SW |
| A. aff. vote sp.1 | Parque Nacional Nascentes do Lago Jari, Brazil | -4.832444 | -61.002528 | MH270030 | MH269840 | MH270242 | INPA-H 27417 | Paratype | SW |
| A. aff. vote sp.1 | Parque Nacional Nascentes do Lago Jari, Brazil | -4.832444 | -61.002528 | MH270031 | MH269841 | MH270243 | INPA-H 27418 | Paratype | SW |
| A. aff. vote sp.1 | Parque Nacional Nascentes do Lago Jari, Brazil | -4.832444 | -61.002528 | MH270032 | MH269842 | MH270244 | INPA-H 27419 | Paratype | SW |
| A. aff. vote sp.1 | Parque Nacional Nascentes do Lago Jari, Brazil | -4.832444 | -61.002528 | MH270033 | MH269843 | MH270245 | INPA-H 27426 | Paratype | SW |
| A. aff. vote sp.1 | Parque Nacional Nascentes do Lago Jari, Brazil | -4.832444 | -61.002528 | KF433970 | MH269844 | MH270246 | INPA-H 28720 | Paratype | SW |
| A. aff. vote sp.1 | Parque Nacional Nascentes do Lago Jari, Brazil | -4.832444 | -61.002528 | KF433971 | MH269845 | MH270247 | INPA-H 28722 | Paratype | SW |
| A. aff. vote sp.1 | Parque Nacional Nascentes do Lago Jari, Brazil | -4.832444 | -61.002528 | MH270034 | MH269846 | MH270248 | INPA-H 27421 | Paratype | SW |
| A. aff. vote sp.1 | Parque Nacional Nascentes do Lago Jari, Brazil | -4.832444 | -61.002528 | MH270035 | MH269847 | MH270249 | INPA-H 27423 | Paratype | SW |
| A. aff. vote sp.1 | Parque Nacional Nascentes do Lago Jari, Brazil | -4.832444 | -61.002528 | MH270036 | MH269848 | MH270250 | INPA-H 27425 | Paratype | SW |
| A. aff. vote sp.1 | Parque estadual Matupiri, Brazil | -5.014444 | -61.270556 | MH270022 | MH269832 | MH270234 | INPA-H 31882 |  | SW |
| A. aff. vote sp.1 | Parque estadual Matupiri, Brazil | -5.014444 | -61.270556 | MH270023 | MH269833 | MH270235 | INPA-H 31874 |  | SW |
| A. aff. vote sp.1 | Parque estadual Matupiri, Brazil | -5.014444 | -61.270556 | MH270024 | MH269834 | MH270236 | INPA-H 31880 |  | SW |
| A. aff. vote sp.1 | Parque estadual Matupiri, Brazil | -5.014444 | -61.270556 | MH270025 | MH269835 | MH270237 | INPA-H 31884 |  | SW |
| A. aff. vote sp.1 | Parque estadual Matupiri, Brazil | -5.014444 | -61.270556 | MH270026 | MH269836 | MH270238 | INPA-H 31870 |  | SW |
| A. aff. vote sp.1 | Parque estadual Matupiri, Brazil | -5.014444 | -61.270556 | MH270027 | MH269837 | MH270239 | INPA-H 31866 |  | SW |
| A. aff. vote sp.1 | Parque estadual Matupiri, Brazil | -5.014444 | -61.270556 | MH270028 | MH269838 | MH270240 | INPA-H 31873 |  | SW |
| A. aff. vote sp.1 | Manaquiri, Amazonas, Brazil | -5.456944 | -62.877222 | MH270019 | MH269829 | MH270231 | INPA-H 35535 |  | SW |
| A. aff. vote sp.1 | Manaquiri, Amazonas, Brazil | -5.456944 | -62.877222 | MH270020 | MH269830 | MH270232 | INPA-H 35536 |  | SW |
| A. aff. vote sp.2 | Manaquiri, Amazonas, Brazil | -5.456944 | -62.877222 | MH270021 | MH269831 | MH270233 | INPA-H 35537 |  | SW |
| A. aff. vote sp.2 | Tapauá, Purus River, Amazonas, Brazil | -5.693472 | -63.199250 | MH270037 | MH269849 | MH270251 | INPA-H 35551 |  | SW |
| A. aff. vote sp.2 | Tapauá, Purus River, Amazonas, Brazil | -5.693472 | -63.199250 | MH270038 | MH269850 | MH270252 | INPA-H 35552 |  | SW |
| A. aff. vote sp.2 | Tapauá, Purus River, Amazonas, Brazil | -5.693472 | -63.199250 | MH270039 | MH269851 | MH270253 | INPA-H 35553 |  | SW |
| A. vote | Aripuanã River, Amazonas, Brazil | -9.166700 | -60.616700 | MH270006 | MH269806 | MH270209 | INPA-H 35540 |  | SW |
| A. vote | Aripuanã River, Amazonas, Brazil | -9.166700 | -60.616700 | MH270007 | MH269807 | MH270210 | INPA-H 35541 |  | SW |
| A. vote | Aripuanã River, Amazonas, Brazil | -9.166700 | -60.616700 | MH270008 | MH269808 | MH270211 | INPA-H 35542 |  | SW |
| A. vote | Aripuanã River, Amazonas, Brazil | -9.166700 | -60.616700 | MH270009 | MH269809 | MH270212 | INPA-H 35543 |  | SW |
| A. vote | Aripuanã River, Amazonas, Brazil | -9.166700 | -60.616700 | MH270010 | MH269810 | MH270213 | INPA-H 35544 |  | SW |
| A. bokermanni | Juruti, Pará, Brazil | -2.216199 | -56.095978 | MH270081 | MH269883 | MH270284 | INPA-H 31863 |  | SW |
| A. bokermanni | Juruti, Pará, Brazil | -2.216199 | -56.095978 | KF433975 | MH269884 | MH270285 | INPA-H 31864 |  | SW |
| A. bokermanni | Juriti, Pará, Brazil | -2.216199 | -56.095978 | KF433976 | MH269885 | MH270286 | INPA-H 31861 |  | SW |
| A. bokermanni | Juriti, Pará, Brazil | -2.216199 | -56.095978 | KF433976 | MH269885 | MH270287 | INPA-H 31862 |  | SW |
| A. bokermanni | Autazes, Amazonas Brazil | -3.727288 | -59.132792 | MH270011 | MH269811 | MH270214 | INPA-H 35529 |  | SW |
| A. bokermanni | Autazes, Amazonas Brazil | -3.727288 | -59.132792 | MH270012 | MH269812 | MH270215 | INPA-H 35530 |  | SW |
| A. bokermanni | Autazes, Amazonas Brazil | -3.727288 | -59.132792 | MH270013 | MH269813 | MH270216 | INPA-H 35531 |  | SW |
| A. bokermanni | Autazes, Amazonas Brazil | -3.727288 | -59.132792 | MH270014 | MH269814 | MH270217 | INPA-H 35532 |  | SW |
| A. bokermanni | Autazes, Amazonas Brazil | -3.727288 | -59.132792 | MH270015 | MH269815 | MH270218 | INPA-H 35528 |  | SW |
| A. bokermanni | Autazes, Amazonas Brazil | -3.727288 | -59.132792 | MH270016 | MH269816 | MH270219 | INPA-H 35529 |  | SW |
| A. bokermanni | Autazes, Amazonas Brazil | -3.727288 | -59.132792 | MH270017 | MH269817 | MH270220 | INPA-H 35530 |  | SW |
| A. bokermanni | Autazes, Amazonas Brazil | -3.727288 | -59.132792 | MH270018 | MH269818 | MH270221 | INPA-H 35531 |  | SW |
| A. bokermanni | Pacamari, Abacaxis River, Amazonas, Brazil | -4.596944 | -58.220556 | JN867562 |  | JN867535 | MTR12679 |  | SW |
| A. bokermanni | Pacamari, Abacaxis River, Amazonas, Brazil | -4.596944 | -58.220556 | JN867563 |  | JN867536 | MTR13037 |  | SW |
| A. bokermanni | Pacamari, Abacaxis River, Amazonas, Brazil | -4.596944 | -58.220556 | JN867561 |  | JN867534 | MTR13036 |  | SW |
| A. xinguensis sp_nov. | Fazenda Paraiso, Xingu River, Pará, Brazil | -3.161694 | -52.128306 | MH270070 | MH269872 | MH270273 | INPA–H 35472 |  | SW |
| A. xinguensis sp_nov. | Fazenda Paraiso, Xingu River, Pará, Brazil | -3.161694 | -52.128306 | MH270071 | MH269873 | MH270274 | INPA–H 35471 |  | SW |
| A. xinguensis sp_nov. | Fazenda Paraiso, Xingu River, Pará, Brazil | -3.161694 | -52.128306 | MH270072 | MH269874 | MH270275 | INPA–H 35493 |  | SW |
| A. xinguensis sp_nov. | PDS Virola Jatoba, Xingu River, Pará, Brazil | -3.168333 | -51.298389 | MH270073 | MH269875 | MH270276 | INPA–H 35484 |  | SW |
| A. xinguensis sp_nov. | PDS Virola Jatoba, Xingu River, Pará, Brazil | -3.168333 | -51.298389 | MH270074 | MH269876 | MH270277 | INPA–H 35485 |  | SW |
| A. xinguensis sp_nov. | PDS Virola Jatoba, Xingu River, Pará, Brazil | -3.168333 | -51.298389 | MH270075 | MH269877 | MH270278 | INPA–H 35473 |  | SW |
| A. xinguensis sp_nov. | PDS Virola Jatoba, Xingu River, Pará, Brazil | -3.168333 | -51.298389 | MH270076 | MH269878 | MH270279 | INPA–H 35474 |  | SW |
| A. xinguensis sp_nov. | PDS Virola Jatoba, Xingu River, Pará, Brazil | -3.168333 | -51.298389 | MH270077 | MH269879 | MH270280 | INPA–H 35490 |  | SW |
| A. xinguensis sp_nov. | PDS Virola Jatoba, Xingu River, Pará, Brazil | -3.168333 | -51.298389 | MH270078 | MH269880 | MH270281 | INPA–H 35483 |  | SW |
| A. xinguensis sp_nov. | Ramal dos Cocos, Xingu River, Pará, Brazil | -2.576944 | -51.830639 | MH270079 | MH269881 | MH270282 | INPA–H 35486 |  | SW |
| A. xinguensis sp_nov. | Ramal dos Cocos, Xingu River, Pará, Brazil | -2.576944 | -51.830639 | MH2700780 | MH269882 | MH270283 | INPA–H 35487 |  | SW |
| A. sp2 | Flona Tapajos, Tapajos River, Pará, Brazil | -5.764881 | -57.270049 | MH270041 | MH269854 | MH270254 | UFPA AA 607 |  | SW |
| A. sp2 | Flona Tapajos, Tapajos River, Pará, Brazil | -3.047654 | -54.979858 | MH270042 | MH269852 | MH270255 | UFPA AA 608 |  | SW |
| A. sp2 | Flona Tapajos, Tapajos River, Pará, Brazil | -5.764881 | -57.270049 | MH270043 | MH269853 | MH270256 | UFPA AA 609 |  | SW |
| A. sp3 | Jatobá, Tapajos River, Pará, Brazil | -5 .245208 | -56 .909929 | MH270044 | MH269819 | MH270221 | UFPA AA 562 |  | SW |
| A. sp3 | Jatobá, Tapajos River, Pará, Brazil | -5 .245208 | -56 .909929 | MH270045 | MH269820 | MH270222 | UFPA AA 563 |  | SW |
| A. sp3 | Vila mamãe Maria, Tapajos River, Pará, Brazil | -5 .738375 | -57 .355460 | MH270046 | MH269821 | MH270223 | UFPA AA 564 |  | SW |
| A. sp3 | Vila mamãe Maria, Tapajos River, Pará, Brazil | -5 .738375 | -57 .355460 | MH270047 | MH269822 | MH270224 | UFPA AA 568 |  | SW |
| A. sp3 | Ponta do Bacabalzinho, Tapajos River, Pará, Brazil | -5 .764881 | -57 .270049 | MH270048 | MH269823 | MH270225 | UFPA AA 573 |  | SW |
| A. sp3 | Preciosa, Tapajos River, Pará, Brazil | -6 .126882 | -57 .587733 | MH270049 | MH269824 | MH270226 | UFPA AA 580 |  | SW |
| A. sp3 | Jatobá, Tapajos River, Pará, Brazil | -5 .245208 | -56 .909929 | MH270050 | MH269825 | MH270227 | UFPA AA 585 |  | SW |
| A. sp3 | Jatobá, Tapajos River, Pará, Brazil | -5 .245208 | -56 .909929 | MH270051 | MH269826 | MH270228 | UFPA AA 586 |  | SW |
| A. sp3 | Vila mamãe Maria, Tapajos River, Pará, Brazil | -5 .738375 | -57 .355460 | MH270052 | MH269827 | MH270229 | UFPA AA 598 |  | SW |
| A. sp3 | Vila mamãe Maria, Tapajos River, Pará, Brazil | -5 .738375 | -57 .355460 | MH270053 | MH269828 | MH270230 | UFPA AA 599 |  | SW |
| A. sp3 | Vila mamãe Maria, Tapajos River, Pará, Brazil | -5 .738375 | -57 .355460 | MH270054 | MH269855 | MH270257 | UFPA AA 600 |  | SW |
| A. sp3 | Fortaleza, Tapajos River, Pará, Brazil | -6 .100396 | -57 .662726 | MH270055 | MH269856 | MH270258 | UFPA AA 557 |  | SW |
| A. sp3 | Fortaleza, Tapajos River, Pará, Brazil | -6 .100396 | -57 .662726 | MH270056 | MH269857 | MH270259 | UFPA AA 558 |  | SW |
| A. sp3 | Fortaleza, Tapajos River, Pará, Brazil | -6 .100396 | -57 .662726 | MH270057 | MH269858 | MH270260 | UFPA AA 577 |  | SW |
| A. sp3 | Fortaleza, Tapajos River, Pará, Brazil | -6 .100396 | -57 .662726 | MH270058 | MH269859 | MH270261 | UFPA AA 581 |  | SW |
| A. sp3 | Vila mamãe Maria, Tapajos River, Pará, Brazil | -5 .738375 | -57 .355460 | MH270059 | MH269860 | MH270262 | UFPA AA 590 |  | SW |
| A. sp3 | Vila mamãe Maria, Tapajos River, Pará, Brazil | -5 .738375 | -57 .355460 | MH270060 | MH269861 | MH270263 | UFPA AA 591 |  | SW |
| A. sp3 | Vila mamãe Maria, Tapajos River, Pará, Brazil | -5 .738375 | -57 .355460 | MH270061 | MH269862 | MH270264 | UFPA AA 592 |  | SW |
| A. sp3 | Vila mamãe Maria, Tapajos River, Pará, Brazil | -5 .738375 | -57 .355460 | MH270062 | MH269863 | MH270265 | UFPA AA 593 |  | SW |
| A. sp3 | Vila mamãe Maria, Tapajos River, Pará, Brazil | -5 .738375 | -57 .355460 | MH270063 | MH269864 | MH270266 | UFPA AA 594 |  | SW |
| A. sp3 | Vila mamãe Maria, Tapajos River, Pará, Brazil | -5 .738375 | -57 .355460 | MH270064 | MH269865 | MH270267 | UFPA AA 595 |  | SW |
| A. sp3 | Vila mamãe Maria, Tapajos River, Pará, Brazil | -5 .738375 | -57 .355460 | MH270065 | MH269866 | MH270268 | UFPA AA 569 |  | SW |
| A. sp3 | Vila mamãe Maria, Tapajos River, Pará, Brazil | -5 .738375 | -57 .355460 | MH270066 | MH269867 | MH270269 | UFPA AA 570 |  | SW |
| A. sp3 | Vila mamãe Maria, Tapajos River, Pará, Brazil | -5 .738375 | -57 .355460 | MH270067 | MH269868 | MH270270 | UFPA AA 571 |  | SW |
| A. sp3 | Vila mamãe Maria, Tapajos River, Pará, Brazil | -5 .738375 | -57 .355460 | MH270068 | MH269869 | MH270271 | UFPA AA 572 |  | SW |
| A. sp3 | Vila mamãe Maria, Tapajos River, Pará, Brazil | -5 .738375 | -57 .355460 | MH270069 | MH269870 | MH270272 | UFPA AA 596 |  | SW |
| A. sp3 | Vila mamãe Maria, Tapajos River, Pará, Brazil | -5 .738375 | -57 .355460 |  | MH269871 |  | UFPA AA 597 |  | SW |
| A. matses | Requena, Loreto, Peru | -5.042500 | -73.833889 | MH269984 | MH269776 | MH270179 | MZUNAP 928 | Paratype | SW |
| A. matses | Jenaro Herrera, Loreto, Peru | -4.898469 | -73.618231 | MH269985 | MH269777 | MH270180 | MZUNAP 929 | Paratype | SW |
| A. matses | Jenaro Herrera, Loreto, Peru | -4.898469 | -73.618231 | MH269986 | MH269778 | MH270181 | MZUNAP 930 | Paratype | SW |
| A. matses | Jenaro Herrera, Loreto, Peru | -4.898469 | -73.618231 | MH269987 | MH269779 | MH270182 | MZUNAP 931 | Paratype | SW |
| A. matses | Jenaro Herrera, Loreto, Peru | -4.898469 | -73.618231 | MH269988 | MH269780 | MH270183 | MZUNAP 933 | Paratype | SW |
| A. matses | Jenaro Herrera, Loreto, Peru | -4.898469 | -73.618231 | MH269989 | MH269781 | MH270184 | MZUNAP 955 | Paratype | SW |
| A. matses | Jenaro Herrera, Loreto, Peru | -4.898469 | -73.618231 | MH269990 | MH269782 | MH270185 | MZUNAP 950 | Paratype | SW |
| A. matses | Jenaro Herrera, Loreto, Peru | -4.898469 | -73.618231 | MH269991 | MH269783 | MH270186 | MZUNAP 937 | Paratype | SW |
| A. matses | Jenaro Herrera, Loreto, Peru | -4.898469 | -73.618231 | MH269992 | MH269784 | MH270187 | MZUNAP 939 | Paratype | SW |
| A. matses | Jenaro Herrera, Loreto, Peru | -4.898469 | -73.618231 | MH269993 | MH269785 | MH270188 | MZUNAP 941 | Paratype | SW |
| A. matses | Jenaro Herrera, Loreto, Peru | -4.898469 | -73.618231 | MH269994 | MH269786 | MH270189 | MZUNAP 942 | Paratype | SW |
| A. matses | Jenaro Herrera, Loreto, Peru | -4.898469 | -73.618231 | MH269995 | MH269787 | MH270190 | MZUNAP 949 | Paratype | SW |
| A. matses | Nuevo Salvador, Loreto, Peru | -5.042500 | -73.833889 | KF681680 | MH269788 | MH270191 | MZUNAP 936 | Paratype | SW |
| A. matses | Nuevo Salvador, Loreto, Peru | -5.042500 | -73.833889 | KF681681 | MH269789 | MH270192 | MZUNAP 941 | Paratopotype | SW |
| A. matses | Nuevo Salvador, Loreto, Peru | -5.042500 | -73.833889 | KF681682 | MH269790 | MH270193 | MZUNAP 934 | Paratopotype | SW |
| A. matses | Nuevo Salvador, Loreto, Peru | -5.042500 | -73.833889 | KF681683 | MH269791 | MH270194 | MZUNAP 958 | Paratopotype | SW |
| A. matses | Nuevo Salvador, Loreto, Peru | -5.042500 | -73.833889 | KF681684 | MH269792 | MH270195 | MZUNAP 922 | Paratopotype | SW |
| A. matses | Nuevo Salvador, Loreto, Peru | -5.042500 | -73.833889 | KF681685 | MH269793 | MH270196 | MZUNAP 923 | Paratopotype | SW |
| A. matses | Nuevo Salvador, Loreto, Peru | -5.042500 | -73.833889 | MH269986 | MH269794 | MH270197 | MZUNAP 934 | Paratopotype | SW |
| A. matses | Nuevo Salvador, Loreto, Peru | -5.042500 | -73.833889 | MH269987 | MH269795 | MH270198 | MZUNAP 955 | Paratopotype | SW |
| A. matses | Nuevo Salvador, Loreto, Peru | -5.042500 | -73.833889 | MH269988 | MH269796 | MH270199 | MZUNAP 940 | Paratopotype | SW |
| A. matses | Nuevo Salvador, Loreto, Peru | -5.042500 | -73.833889 | MH269989 | MH269797 | MH270200 | MZUNAP 948 | Paratopotype | SW |
| A. matses | Nuevo Salvador, Loreto, Peru | -5.042500 | -73.833889 | MH269990 | MH269798 | MH270201 | MZUNAP 943 | Paratopotype | SW |
| A. aff. matses sp. 1 | Carauari, Jurua River, Brazil | -4.863162 | -66.976975 | MH270001 | MH269799 | MH270202 | MZUNAP 952 |  | SW |
| A. aff. matses sp. 1 | Carauari, Jurua River, Brazil | -4.863162 | -66.976975 | MH270002 | MH269800 | MH270203 | MZUNAP 953 |  | SW |
| A. aff. matses sp. 1 | Carauari, Jurua River, Brazil | -4.863162 | -66.976975 | MH270003 | MH269801 | MH270204 | MZUNAP 932 |  | SW |
| A. javierbustamantei | Quebrada Guacamayo, Madre de Dios, Peru | -12.906806 | -69.992417 | KR905184 | MH269802 | MH270205 | MHNC 8331 | Holotype | SW |
| A. javierbustamantei | Quebrada Guacamayo, Madre de Dios, Peru | -12.906806 | -69.992417 | KR905185 | MH269803 | MH270206 | MHNC 8363 | Paratype | SW |
| A. moisesii sp.nov. | Panguana, Rio Yuyaapichis, Huanuco, Peru | -8 .57 | -75 .48 | DQ502120 | DQ502120 | DQ502120 | MJH 7095 |  | SW |
| A. moisesii sp.nov | Acre, Brazil | -7 .42 | -73 .65 | MH270004 | MH269805 | MH270207 | PRMS 0404 |  | SW |
| A. moisesii sp.nov | Acre, Brazil | -8 .57 | -75 .48 | MH270005 | MH269804 | MH270208 | PRMS 0420 |  | SW |
| A. amazonicola | Fundo Zamora, Loreto, Peru | -3.966111 | -73.418611 | MH269931 | MH269714 | MH270116 | MZUNAP 908 | Paratype | NW |
| A. amazonicola | Fundo Zamora, Loreto, Peru | -3.966111 | -73.418611 | MH269932 | MH269715 | MH270117 | MZUNAP 924 | Paratype | NW |
| A. amazonicola | Fundo Zamora, Loreto, Peru | -3.966111 | -73.418611 | MH269933 | MH269716 | MH270118 | MZUNAP 886 | Paratype | NW |
| A. amazonicola | Fundo Zamora, Loreto, Peru | -3.966111 | -73.418611 | MH269934 | MH269717 | MH270119 | MZUNAP 900 | Paratype | NW |
| A. amazonicola | Fundo Zamora, Loreto, Peru | -3.966111 | -73.418611 | MH269935 | MH269718 | MH270120 | MZUNAP 888 | Paratype | NW |
| A. amazonicola | Fundo Zamora, Loreto, Peru | -3.966111 | -73.418611 | MH269936 | MH269719 | MH270121 | MZUNAP 902 | Paratype | NW |
| A. amazonicola | Fundo Zamora, Loreto, Peru | -3.966111 | -73.418611 | MH269937 | MH269720 | MH270122 | MZUNAP 887 | Paratype | NW |
| A. amazonicola | Fundo Zamora, Loreto, Peru | -3.966111 | -73.418611 | MH269938 | MH269721 | MH270123 | MZUNAP 920 | Paratype | NW |
| A. amazonicola | Fundo Zamora, Loreto, Peru | -3.966111 | -73.418611 | MH269939 | MH269722 | MH270124 | MZUNAP 905 | Paratype | NW |
| A. amazonicola | Fundo UNAP, Loreto, Peru | -4.035223 | -73.450595 | MH269940 | MH269723 | MH270125 | MZUNAP 242 | Paratype | NW |
| A. amazonicola | Nauta, Loreto, Peru | -4.569167 | -73.723611 | MH269941 | MH269724 | MH270126 | MZUNAP 909 | Paratype | NW |
| A. amazonicola | Puerto Almendras, Loreto, Peru | -3.828056 | -73.368611 | MH269942 | MH269725 | MH270127 | MZUNAP 889 | Paratype | NW |
| A. amazonicola | Puerto Almendras, Loreto, Peru | -3.828056 | -73.368611 | MH269943 | MH269726 | MH270128 | MZUNAP 914 | Paratype | NW |
| A. amazonicola | Puerto Almendras, Loreto, Peru | -3.828056 | -73.368611 | MH269944 | MH269727 | MH270129 | MZUNAP 913 | Paratype | NW |
| A. amazonicola | Puerto Almendras, Loreto, Peru | -3.828056 | -73.368611 | MH269945 | MH269728 | MH270130 | MZUNAP 916 | Paratype | NW |
| A. amazonicola | Puerto Almendras, Loreto, Peru | -3.828056 | -73.368611 | MH269946 | MH269729 | MH270131 | MZUNAP 911 | Paratype | NW |
| A. amazonicola | Puerto Almendras, Loreto, Peru | -3.828056 | -73.368611 | MH269947 | MH269730 | MH270132 | MZUNAP 910 | Paratype | NW |
| A. amazonicola | Puerto Almendras, Loreto, Peru | -3.828056 | -73.368611 | MH269948 | MH269731 | MH270133 | MZUNAP 889 | Paratype | NW |
| A. amazonicola | Puerto Almendras, Loreto, Peru | -3.828056 | -73.368611 |  | MH269732 | MH270134 | MZUNAP 913 | Paratype | NW |
| A. amazonicola | Puerto Almendras, Loreto, Peru | -3.828056 | -73.368611 | KF681668 | MH269733 | MH270135 | MZUNAP 901 | Holotype | NW |
| A. amazonicola | Puerto Almendras, Loreto, Peru | -3.828056 | -73.368611 | KF681669 | MH269734 | MH270136 | MZUNAP 915 | Paratopotype | NW |
| A. amazonicola | Puerto Almendras, Loreto, Peru | -3.828056 | -73.368611 | KF681670 | MH269735 | MH270137 | MZUNAP 906 | Paratopotype | NW |
| A. amazonicola | Puerto Almendras, Loreto, Peru | -3.828056 | -73.368611 | KF681671 | MH269736 | MH270138 | MZUNAP 907 | Paratopotype | NW |
| A. amazonicola | Puerto Almendras, Loreto, Peru | -3.828056 | -73.368611 | KF681672 | MH269737 | MH270139 | MZUNAP 110 | Paratopotype | NW |
| A. amazonicola | Puerto Almendras, Loreto, Peru | -3.828056 | -73.368611 | KF681673 | MH269738 | MH270140 | MZUNAP 917 | Paratopotype | NW |
| A. siona sp. nov. | Pankins, Morona Santiago, Ecuador | -2.80395 | -77.49367 | MH270111 | MH269926 | MH270326 | QCAZ 53279 |  | NW |
| A. siona sp. nov. | Nuevo Israel, Morona Santiago, Ecuador | -2.16500 | -77.90292 | MH270112 | MH269927 | MH270327 | QCAZ 46440 |  | NW |
| A. siona sp. nov. | Pankins, Morona Santiago, Ecuador | -2.803950 | -77.493670 | MH270113 | MH269928 | MH270328 | QCAZ 46430 |  | NW |
| A. siona sp. nov. | Parque Yasuni, Ecuador | -0.666944 | -76.442500 | MH270114 | MH269929 | MH270329 | QCAZ 61068 |  | NW |
| A. siona sp. nov. | Parque Yasuni, Ecuador | -0.941944 | -75.905000 | MH270115 | MH269930 | MH270330 | QCAZ 52823 |  | NW |
| A. siona sp. nov. | Rio Lagarto Cocha, Peru |  |  | AY326000 | AY326000 | AY326000 | USNM 520905 |  | NW |
| A. siona sp. nov. | Equador |  |  | DQ158262 | DQ158262 | DQ158262 | QCAZ833 |  | NW |
| A. minuta | Taracuá, Amazonas, Brazil | 0.094661 | -68.466130 | KF792834 | MH269760 | MH270163 | INPA-H 32729 | Topotype | NW |
| A. minuta | Taracuá, Amazonas, Brazil | 0.094661 | -68.466130 | KF792835 | MH269761 | MH270164 | INPA-H 32730 | Topotype | NW |
| A. minuta | Taracuá, Amazonas, Brazil | 0.094661 | -68.466130 | MH269971 | MH269762 | MH270165 | INPA-H 32732 | Topotype | NW |
| A. minuta | Taracuá, Amazonas, Brazil | 0.094661 | -68.466130 | MH269972 | MH269763 | MH270166 | INPA-H 32733 | Topotype | NW |
| A. minuta | Taracuá, Amazonas, Brazil | 0.094661 | -68.466130 | MH269973 | MH269764 | MH270167 | INPA-H 32738 | Topotype | NW |
| A. minuta | Taracuá, Amazonas, Brazil | 0.094661 | -68.466130 | MH269974 | MH269765 | MH270168 | INPA-H 32737 | Topotype | NW |
| A. minuta | Taracuá, Amazonas, Brazil | 0.094661 | -68.466130 | MH269975 | MH269766 | MH270169 | INPA-H 32730 | Topotype | NW |
| A. minuta | Taracuá, Amazonas, Brazil | 0.094661 | -68.466130 | MH269976 | MH269767 | MH270170 | INPA–H 32725 | Topotype | NW |
| A. minuta | Taracuá, Amazonas, Brazil | 0.094661 | -68.466130 | MH269977 | MH269768 | MH270171 | INPA–H 32726 | Topotype | NW |
| A. minuta | Taracuá, Amazonas, Brazil | 0.094661 | -68.466130 | MH269978 | MH269769 | MH270172 | INPA–H 32728 | Topotype | NW |
| A. minuta | Taracuá, Amazonas, Brazil | 0.094661 | -68.466130 | MH269979 | MH269770 | MH270173 | INPA–H 32729 | Topotype | NW |
| A. minuta | Taracuá, Amazonas, Brazil | 0.094661 | -68.466130 | MH269980 | MH269771 | MH270174 | INPA–H 32730 | Topotype | NW |
| A. minuta | Taracuá, Amazonas, Brazil | 0.094661 | -68.466130 | MH269981 | MH269772 | MH270175 | INPA–H 32732 | Topotype | NW |
| A. minuta | Taracuá, Amazonas, Brazil | 0.094661 | -68.466130 | MH269982 | MH269773 | MH270176 | INPA–H 32733 | Topotype | NW |
| A. minuta | Taracuá, Amazonas, Brazil | 0.094661 | -68.466130 | MH269983 | MH269774 | MH270177 | INPA–H 32735 | Topotype | NW |
| A. minuta | Taracuá, Amazonas, Brazil | 0.094661 | -68.466130 |  | MH269775 | MH270178 | INPA–H 32740 | Topotype | NW |
| A. minuta | São Gabriel da Cachoeira, Amazonas, Brazil | -0.163433 | -66.980272 | MH269966 | MH269755 | MH270158 | INPA-H 32728 |  | NW |
| A. minuta | São Gabriel da Cachoeira, Amazonas, Brazil | -0.163433 | -66.980272 | MH269967 | MH269756 | MH270159 | INPA-H 32734 |  | NW |
| A. minuta | São Gabriel da Cachoeira, Amazonas, Brazil | -0.163433 | -66.980272 | MH269968 | MH269757 | MH270160 | INPA-H 32731 |  | NW |
| A. minuta | São Gabriel da Cachoeira, Amazonas, Brazil | -0.163433 | -66.980272 | MH269969 | MH269758 | MH270161 | INPA-H 32735 |  | NW |
| A. minuta | São Gabriel da Cachoeira, Amazonas, Brazil | -0.163433 | -66.980272 | MH269970 | MH269759 | MH270162 | INPA-H 32741 |  | NW |
| A. aff. minuta sp1 | Japura River, Amazonas, Brazil | -1.814577 | -68.981414 | MH269949 | MH269739 | MH270141 | INPA-H 35494 |  | NW |
| A. aff. minuta sp1 | Japura River, Amazonas, Brazil | -1.814577 | -68.981414 | MH269950 | MH269740 | MH270142 | INPA-H 35495 |  | NW |
| A. aff. minuta sp1 | Japura River, Amazonas, Brazil | -1.814577 | -68.981414 | MH269951 | MH269741 | MH270143 | INPA-H 35496 |  | NW |
| A. aff..minuta sp1 | Japura River, Amazonas, Brazil | -1.814577 | -68.981414 | MH269952 | MH269742 | MH270144 | INPA-H 35497 |  | NW |
| A. aff. minuta sp1 | Japura River, Amazonas, Brazil | -1.814577 | -68.981414 | MH269953 | MH269743 | MH270145 | INPA-H 35498 |  | NW |
| A. aff..minuta sp1 | Japura River, Amazonas, Brazil | -1.814577 | -68.981414 | MH269954 | MH269744 | MH270146 | INPA-H 35499 |  | NW |
| A. aff. minuta sp1 | Japura River, Amazonas, Brazil | -1.814577 | -68.981414 | MH269955 | MH269745 | MH270147 | INPA-H 35500 |  | NW |
| A. aff..minuta sp1 | Japura River, Amazonas, Brazil | -1.814577 | -68.981414 | MH269956 | MH269746 | MH270148 | INPA-H 35501 |  | NW |
| A. aff..minuta sp1 | Japura River, Amazonas, Brazil | -1.814577 | -68.981414 | MH269957 | MH269747 | MH270149 | INPA-H 35502 |  | NW |
| A. aff..minuta sp1 | Japura River, Amazonas, Brazil | -1.814577 | -68.981414 | MH269958 | MH269748 | MH270150 | INPA-H 35503 |  | NW |
| A. aff. minuta sp1 | Japura River, Amazonas, Brazil | -1.814577 | -68.981414 | MH269959 | MH269749 | MH270151 | INPA-H 35504 |  | NW |
| A. aff..minuta sp1 | Japura River, Amazonas, Brazil | -1.814577 | -68.981414 | MH269960 | MH269750 | MH270152 | INPA-H 35494 |  | NW |
| A. aff.. minuta sp1 | Japura River, Amazonas, Brazil | -1.814577 | -68.981414 | MH269961 | MH269751 | MH270153 | INPA-H 35495 |  | NW |
| A. aff.. minuta sp1 | Japura River, Amazonas, Brazil | -1.814577 | -68.981414 | MH269962 | MH269752 | MH270154 | INPA-H 35496 |  | NW |
| A. aff.. minuta sp1 | Japura River, Amazonas, Brazil | -1.814577 | -68.981414 | MH269963 | MH269753 | MH270155 | INPA-H 35497 |  | NW |
| A. aff.. minuta sp1 | Japura River, Amazonas, Brazil | -1.814577 | -68.981414 | MH269964 | MH269754 | MH270156 | INPA-H 35498 |  | NW |

Supplementary Table S2b List of non-Amazophrynella taxa used in this study, with GenBank accession numbers.

| Sample | Locality | 16S | 12S | Voucher number |
| --- | --- | --- | --- | --- |
| Rhinella sp. aff. margaritifera | Madre de dios, Tambopata, Peru | JN867547 | JN867573 | ROM40103 |
| Rhinella sp. aff. margaritifera | APM Manso, Chapada dos Guimarães, Mato Grosso, Brazil | JN867549 | JN867575 | AF727; 5337 |
| Rhinella hoogmoedi | Serra Bonita, Camacan, Bahia, Brazil | JN867545 | JN867571 | MTR16199 |
| Rhinella sp. aff. margaritifera | Carolina, Argentina | JN867548 | JN867574 | ESTR00173 |
| Rhinella ocellata | UHE Lajeado, Palmas, Tocantins, Brazil | JN867546 | JN867572 | LAJ210 |
| Melanophryniscus stelzneri | Parque Nacional San Rafael, Paraguay | DQ158421 | DQ158421 | KU28907 |
| Oreophrynella sp. | Mazaruni-Potaro District, Mount Ayanganna, Guyana | DQ158422 | DQ158422 | ROM39649 |
| Atelopus peruensis | Cajamarca, Peru | DQ158419 | DQ158419 | KU211632 |
| Nannophryne variegatus | Puerto Edén, Isla Wellington, Chile | DQ158494 | DQ158494 | IZUA 3198 |
| Nannophryne cophotis | Cajamarca, S slope Abra Quilsh, Peru | DQ158446 | DQ158446 | KU211685 |
| Rhaebo nasicus | Mazaruni-Potaro District, Potaro River, Guyana | DQ158477 | DQ158477 | ROM20560 |
| Rhaebo guttatus | Parque Estadual Guajara-Mirim, Roraima, Brazil | DQ158459 | DQ158459 | LSUMZ17418 |
| Peltophryne lemur | Puerto-Rico | DQ158465 | DQ158465 | A. Goebel |
| Ingerophryne galeatus | Khammouane Prov. Nakai Dist. Nakai Nam, Lao | DQ158452 | DQ158452 | FMNH256443 |
| Pseudepidalea viridis | pet trade | DQ283279 | DQ283279 | MNHA168402/MVZ164718 |
| Nectophrynoides tornieri | Tanzania | FJ882815 | FJ882815 | MW1822/BMNH2005.1375 |
| Duttaphryne melanostictus | Ha Tinh, Huang Son Reserve, Rao An region, Vietnan | DQ283333 | DQ283333 | AMNHA161135/AMNH16001 |
| Schismaderma carens | Harare, Zimbabwe | DQ158424 | DQ158424 | MVZ223386 |
| Epidalea calamita | ? | EU938400 | EU938400 | MVZ164859/MVZ186039 |
| Bufo gutturalis | Mumba Village, Tanzania | DQ283436 | DQ283436 | RdS873/CAS214842 |
| Leptophryne borbonica | Borneo, Sabah, Crocker Range, Ulu Kimanis, Malaysia | AB331716 | AB331716 | BORNEENSIS08127/VUB0673 |
| Pedostibes hosii | Danum Valley Research Center, Malaysia | DQ283164 | DQ283164 | FMNH231190/VUB0661 |
| Anaxyrus boreas | CA, San Diego Co., USA | DQ283180 | DQ283180 | RNF2416/MVZ223292 |
| Incilius alvarius | Arizona, Santa Cruz, USA | DQ283269 | DQ283269 | ATH499/USNM320001 |
| Incilius coniferus | Cocle, Parque Nacional El Cop, Panama | DQ283166 | DQ283166 | SIUC6913/KU217480 |
| Rhinella atacamensis | Coquimbo, Chile | DQ158433 | DQ158433 | KU217352 |
| Rhinella marina | Pichincha, Tinalandia, Ecuador | AY680259 | AY680259 | KU202274/KU217482 |
| Rhinella "granulosa" | Southern Rupununi Savanna, Aishalton, Guyana | DQ283332 | DQ283332 | AMNHA139020/AF0093 |
| Rhinella veraguensis | La Paz, Bolivia | DQ158496 | DQ158496 | dlRiva3820 |
| Rhinella (Rhamphophryne) festae | pastaza, Ecuador | DQ158423 | DQ158423 | KU217501 |
| Rhinella nesiotes | La Paz, Bolivia | DQ158478 | DQ158478 | UTA53310 |
| Rhinella ocellata | Peixe, Tocantins, Brazil | DQ158479 | DQ158479 | MZUSP103261 |
| Rhinella sp. aff. margaritifera | Madre de Dios, Peru | DQ158490 | DQ158490 | USNM268828 |
| Rhinella castaneotica | 100 km S Santarem, Para, Brazil | DQ158440 | DQ158440 | LSUMZ17429 |
| Rhinella "dapsilis" | Pichincha, Bosque Protector La Perla, Ecuador | DQ158448 | DQ158448 | QCAZ3509 |
| Pseudopaludicola falcipes | Corrientes, Yapeyu, Argentina | AY843741 | AY843741 | MACN38647/5468 |
| Physalaemus cuvieri/ sp. | Misiones, San Vicente, Argentina | AY843729 | AY843729 | MACN38640/CTMZ05253 |
| D. leucomyxtas | Ilha grande, Brazil | JN867558 | JN867531 | MTR15547 |
| D. leucomyxtas | Ilha grande, Brazil | JN867530 | JN867530 | MTR15548 |
| D. proboscideus | Mata Escura, Brazil | JN867566 | JN867538 | MTR16424 |
| D. proboscideus | Mata Escura, Brazil | JN867539 | JN867539 | MTR17173 |
| D. oreites | Serra das lontras, Brazil | JN867567 | JN867540 | MTR16368 |
| D. carvalhoi | Mata Escura, Brazil | JN867542 | JN867542 | MTR15757 |
| D. carvalhoi | Parna Caparão, Brazil | JN867568 | JN867541 | MTR15755 |
| D. berthalutzae | Treviso, Brazil | JN867551 | JN867524 | CFBH10322 |
| D. brevipollicatus | Parque estadual Carlos Botelho, São Paulo, Brazil |  |  | CFBH19280 |
| D. brevipollicatus | Treviso, São Carlos, Brazil | JN867551 | JN867524 | CFBH10322 |
| D. brevipollicatus | Brazil: Juquitiba, SP | JN867556 | JN867529 | JUQ |
| D. brevipollicatus | Sao Bernardo, São Paulo, Brazil | JN867555 | JN867528 | TH040AF1025 |
| D. brevipollicatus | Estacao Biologica de Boraceia, São Paulo, Brazil | JN867554 | JN867527 | AF1541 |
| D. brevipollicatus | APA, Dona Francisca, Joinville, São Carlos, Brazil |  |  | CFBH19726 |
| D. brevipollicatus | Caucaia do Alto, Reserva do Morro Grande, São Paulo, Brazil | JN867553 | JN867526 | AF1577 |
| D. leucomyxtas | Ilha grande, Brazil | JN867558 | JN867531 | MTR15547 |
| D. leucomyxtas | Ilha grande, Brazil | JN867530 | JN867530 | MTR15548 |
| D. proboscideus | Mata Escura, Brazil | JN867566 | JN867538 | MTR16424 |
| D. proboscideus | Mata Escura, Brazil | JN867539 | JN867539 | MTR17173 |
| D. oreites | Serra das lontras, Brazil | JN867567 | JN867540 | MTR16368 |
| D. carvalhoi | Mata Escura, Brazil | JN867542 | JN867542 | MTR15757 |
| D. carvalhoi | Parna Caparão, Brazil | JN867568 | JN867541 | MTR15755 |
| D. berthalutzae | Treviso, Brazil | JN867551 | JN867524 | CFBH10322 |
| D. brevipollicatus | Parque estadual Carlos Botelho, São Paulo, Brazil |  |  | CFBH19280 |
| D. brevipollicatus | Treviso, São Carlos, Brazil | JN867551 | JN867524 | CFBH10322 |
| D. brevipollicatus | Brazil: Juquitiba, SP | JN867556 | JN867529 | JUQ |
| D. brevipollicatus | Sao Bernardo, São Paulo, Brazil | JN867555 | JN867528 | TH040AF1025 |
| D. brevipollicatus | Estacao Biologica de Boraceia, São Paulo, Brazil | JN867554 | JN867527 | AF1541 |
| D. brevipollicatus | APA, Dona Francisca, Joinville, São Carlos, Brazil |  |  | CFBH19726 |
| D. brevipollicatus | Caucaia do Alto, Reserva do Morro Grande, São Paulo, Brazil | JN867553 | JN867526 | AF1577 |
| D. leucomyxtas | Ilha grande, Brazil | JN867558 | JN867531 | MTR15547 |
| D. leucomyxtas | Ilha grande, Brazil | JN867530 | JN867530 | MTR15548 |
| D. proboscideus | Mata Escura, Brazil | JN867566 | JN867538 | MTR16424 |
| D. proboscideus | Mata Escura, Brazil | JN867539 | JN867539 | MTR17173 |
| D. oreites | Serra das lontras, Brazil | JN867567 | JN867540 | MTR16368 |
