# Supplementary material for: A Pan-Amazonian species delimitation: high species diversity within the genus Amazophrynella (Anura: Bufonidae)
Source: PeerJ. 2018 Jul 9;6:e4941. doi: 10.7717/peerj.4941 (PMC6042491; doi:10.7717/peerj.4941)
Supplement: Supplementary file 3 [file peerj-06-4941-s003.odt]

SUPPORTING INFORMATION

A Pan-Amazonian species delimitation: high species diversity within the genus Amazophrynella (Anura: Bufonidae)

APPENDIX 3 – GEOGRAPHIC DATA FOR ENVIROMENTAL ANALYSES 

Supplementary Table S3a. Data for environmental analysis 

| Lineage | Locality | State/province | Country | Long. | Lat. |
| --- | --- | --- | --- | --- | --- |
| A. minuta | Taracuá | Amazonas | Brazil | -67 .08 | -0 .12 |
| A. minuta | São Gabriel | Amazonas | Brazil | -66 .98 | -0 .16 |
| A. minuta | Lago Cucui | Amazonas | Brazil | -66 .86 | -0 .88 |
| A. minuta | Mitu | Amazonas | Colombia | -70 .48 | 1 .16 |
| A. minuta | Taraira | Amazonas | Colombia | -69 .67 | -0 .90 |
| A .minuta | Estación Biologica Caparu | Amazonas | Colombia | -70 .44 | 0 .45 |
| A .minuta | Uauapes, River | Amazonas | Colombia | -70 .22 | 1 .25 |
| A. minuta | Raudal Rio Cuao | Amazonas | Venezuela | -67 .19 | 4 .51 |
| A. aff. minuta sp1 | Japura, River | Amazonas | Brazil | -69 .98 | -1 .91 |
| A. aff. minuta sp1 | Japura, River | Amazonas | Brazil | -68 .98 | -1 .81 |
| A. siona sp. nov. | Santa Cecilia | Sucumbios | Ecuador | -76 .99 | -0 .08 |
| A. siona sp. nov. | Reserva Yasuni | Orellana | Ecuador | -75 .91 | -0 .94 |
| A. siona sp. nov. | Bataburo Lodge | Pastaza | Ecuador | -76 .72 | -1 .21 |
| A. siona sp. nov. | Cuyabeno | Sucumbios | Ecuador | -76 .17 | -0 .02 |
| A. siona sp. nov. | Pindo | Orellana | Ecuador | -76 .77 | -0 .75 |
| A. siona sp. nov. | Kurintza | Pastaza | Ecuador | -76 .80 | -2 .06 |
| A. siona sp. nov. | Campo Agrooil | Pastaza | Ecuador | -77 .51 | -1 .51 |
| A. siona sp. nov. | Pankins | Morona santiago | Ecuador | -77 .89 | -2 .90 |
| A. siona sp. nov. | Puyo | Morona Santiago | Ecuador | -78 .00 | -1 .41 |
| A. siona sp. nov. | Kapawi Lodge | Pastaza | Ecuador | -76 .86 | -2 .54 |
| A. siona sp. nov. | Lorocachi | Pastaza | Ecuador | -75 .99 | -1 .62 |
| A. amazonicola | Alto Nanay river | Loreto | Peru | -74 .01 | -3 .17 |
| A. amazonicola | Mazan | Loreto | Peru | -73 .12 | -3 .51 |
| A. amazonicola | Cuenca media Nanay | Loreto | Peru | -74 .17 | -3 .82 |
| A. amazonicola | Nauta | Loreto | Peru | -73 .72 | -4 .57 |
| A. amazonicola | Fazenda Zamora | Loreto | Peru | -73 .42 | -3 .97 |
| A. amazonicola | Fundo UNAP | Loreto | Peru | -73 .45 | -4 .04 |
| A. amazonicola | Puerto Almendra | Loreto | Peru | -73 .37 | -3 .83 |
| A. matses | Jurua river | Amazonas | Brazil | -66 .98 | -4 .86 |
| A. matses | Nuevo Salvador | Loreto | Peru | -73 .83 | -5 .04 |
| A. matses | Chonco | Loreto | Peru | -73 .61 | -5 .56 |
| A. matses | Actiame | Loreto | Peru | -73 .16 | -6 .32 |
| A. matses | Jenaro Herrera | Loreto | Peru | -73 .62 | -4 .90 |
| A. matses | Itia Tebu | Loreto | Peru | -73 .76 | -5 .85 |
| A. matses | Requena | Loreto | Peru | -73 .83 | -5 .04 |
| A. javierbustamentei | Tambopata | Madre de Dios | Peru | -69 .58 | -12 .97 |
| A. javierbustamentei | Mapi | Madre de Dios | Peru | -73 .47 | -11 .52 |
| A. javierbustamentei | Tsoroja | Cusco | Peru | -73 .54 | -11 .32 |
| A. javierbustamentei | Camana | Cusco | Peru | -73 .05 | -12 .09 |
| A. javierbustamentei | Inanbari | Madre de Dios | Peru | -70 .38 | -13 .04 |
| A. javierbustamentei | La Pampa | Cusco | Peru | -72 .46 | -12 .67 |
| A. javierbustamentei | Quebrada Guacamayo | Madre de Dios | Peru | -69 .99 | -12 .91 |
| A. javierbustamentei | Panguana | Huanuco | Peru | -74 .79 | -9 .58 |
| A. moisesii sp. nov. | Envira | Amazonas | Brazil | -7 .50 | -73 .60 |
| A. moisesii sp. nov. | Mâncio Lima | Acre | Brazil | -8 .97 | -72 .48 |
| A. moisesii sp. nov. | Parque Nacional Serra do Divisor | Acre | Brazil | -7.410 | -73.652 |
| A. moisesii sp. nov. | Acre | Acre | Brazil | -8 .57 | -75 .48 |
| A. moisesii sp. nov. | Acre | Acre | Brazil | -7 .42 | -73 .65 |
| A. manaos | Campus UFAM | Amazonas | Brazil | -59 .97 | -3 .10 |
| A. manaos | Rio cuieiras | Amazonas | Brazil | -60 .41 | -2 .72 |
| A. manaos | Presidente Figueiredo | Amazonas | Brazil | -60 .04 | -1 .94 |
| A. manaos | Mineração taboca | Amazonas | Brazil | -60 .17 | -0 .72 |
| A. manaos | Zona Franca | Amazonas | Brazil | -60 .12 | -2 .59 |
| A. manaos | Balbina | Amazonas | Brazil | -1 .72 | -59 .19 |
| A. manaos | Figueiredo Road | Amazonas | Brazil | -1 .43 | -60 .45 |
| A. manaos | Taboca Road | Amazonas | Brazil | -0 .85 | -60 .38 |
| A. manaos | Iwokrama Forest Reserve | Iwokrama | Guyana | 3 .74 | -58 .51 |
| A. teko sp. nov. | Trombetas River | Amazonas | Brazil | -1 .43 | -56 .75 |
| A. teko sp. nov. | Amapá | Amapá | Brazil | 0 .23 | -51 .86 |
| A.sp1 | Trombetas River | Amazonas | Brazil | -1 .20 | -56 .67 |
| A.sp1 | Trombetas River | Amazonas | Brazil | -1 .28 | -55 .67 |
| A.sp1 | Cachoeira Porteira | Amazonas | Brazil | -0 .75 | -60 .80 |
| A. teko sp. nov. | Cachoeira Porteira | Amazonas | Brazil | -0 .74 | -59 .80 |
| A. teko sp. nov. | Mitan | Cayene | French Guyana | 2 .63 | -52 .55 |
| A. teko sp. nov. | Alikené | Cayene | French Guyana | 3 .22 | -52 .40 |
| A. teko sp. nov. | Saint Georges | Cayene | French Guyana | 3 .97 | -51 .87 |
| A. teko sp. nov. | Mitaraka Layo | St. Laurent Du Marioni | French Guyana | 2 .24 | -54 .45 |
| A. teko sp. nov. | Flat de la Waki | St. Laurent Du Marioni | French Guyana | 3 .09 | -53 .40 |
| A. xinguensis sp.nov. | Senador Porfirio | Pará | Brazil | -2 .58 | -51 .83 |
| A. xinguensis sp.nov. | Virola Jatobá | Pará | Brazil | -3 .17 | -51 .30 |
| A. xinguensis sp.nov. | Ramal dos cocos | Pará | Brazil | -3 .16 | -52 .13 |
| A. xinguensis sp.nov. | Flona Tapajós | Pará | Brazil | -3 .05 | -54 .98 |
| A. xinguensis sp.nov. | Reserva Tapajós | Pará | Brazil | -3 .33 | -55 .19 |
| A. xinguensis sp.nov. | Flona Jatoba | Pará | Brazil | -5 .76 | -57 .27 |
| A. xinguensis sp.nov. | UHE Jatoba | Pará | Brazil | -6 .13 | -57 .59 |
| A. xinguensis sp.nov. | Itaituba | Pará | Brazil | -4 .66 | -55 .60 |
| A.bokermanni | Abacaxis River | Amazonas | Brazil | -4 .60 | -58 .22 |
| A.bokermanni | Juruti | Amazonas | Brazil | -2 .46 | -56 .05 |
| A.bokermanni | Autazes | Amazonas | Brazil | -3 .80 | -58 .69 |
| A. vote | Fazenda São Nicolau | Mato Grosso | Brazil | -10 .04 | -58 .12 |
| A.vote | Reserva Biológica Jaru | Amazonas | Brazil | -10 .27 | -61 .67 |
| A.vote | Colniza | Amazonas | Brazil | -8 .94 | -59 .07 |
| A.vote | Fazenda Maracatia | Amazonas | Brazil | -9 .36 | -61 .27 |
| A.vote | Aripuanã River | Amazonas | Brazil | -6 .94 | -60 .53 |
| A.vote | Igarapé Açuã | Amazonas | Brazil | -7 .19 | -64 .19 |
| A.vote | Cachoeirinha | Amazonas | Brazil | -5 .49 | -60 .82 |
| A. vote sp1 | Matupiri | Amazonas | Brazil | -5 .01 | -61 .27 |
| A. vote sp1 | Manaquiri | Amazonas | Brazil | -4 .24 | -60 .60 |
| A. vote sp1 | Igapó Açu | Amazonas | Brazil | -4 .75 | -61 .40 |
| A. vote sp1 | Parque Nacional Nascentes do Lago Jari | Amazonas | Brazil | -5 .46 | -62 .88 |
| A. vote sp1 | Purus River | Amazonas | Brazil | -5 .60 | -63 .37 |
