# Supplementary material for: A Pan-Amazonian species delimitation: high species diversity within the genus Amazophrynella (Anura: Bufonidae)
Source: PeerJ. 2018 Jul 9;6:e4941. doi: 10.7717/peerj.4941 (PMC6042491; doi:10.7717/peerj.4941)
Supplement: Supplementary file 4 [file peerj-06-4941-s004.odt]

SUPPORTING INFORMATION

A Pan-Amazonian species delimitation: high species diversity within the genus Amazophrynella (Anura: Bufonidae)

Supplementary Figure S4a Maximum likelihood solution inferred by Poison tree processes (bPTP) model on the best tree solution from Mrbayes. Values in branches are posterior probabilities (values < 0.5 not shown).
