# Supplementary material for: A Pan-Amazonian species delimitation: high species diversity within the genus Amazophrynella (Anura: Bufonidae)
Source: PeerJ. 2018 Jul 9;6:e4941. doi: 10.7717/peerj.4941 (PMC6042491; doi:10.7717/peerj.4941)
Supplement: Supplementary file 5 [file peerj-06-4941-s005.odt]

SUPPORTING INFORMATION

A Pan-Amazonian species delimitation: high species diversity within the genus Amazophrynella (Anura: Bufonidae)

APPENDIX 5 – Divergence time calibration

Supplementary Fig. S5a Phylogenetic relationships of bufonids recovered in BEAST analysis (mitochondrial 16S and 12S). Values at each node represent mean of divergence time. Posterior probability if each node is above the branch. The green color indicates the phylogenetic position of Amazophrynella and their sister taxa Dendrophryniscus
