# Supplementary material for: A Pan-Amazonian species delimitation: high species diversity within the genus Amazophrynella (Anura: Bufonidae)
Source: PeerJ. 2018 Jul 9;6:e4941. doi: 10.7717/peerj.4941 (PMC6042491; doi:10.7717/peerj.4941)
Supplement: Supplementary file 6 [file peerj-06-4941-s007.odt]

SUPPORTING INFORMATION

A Pan-Amazonian species delimitation: integrative methods reveals high species diversity within the genus Amazophrynella (Anura: Bufonidae)

APPENDIX 7 – The Principal component analyses in both clades (eastern and western) revealed a grouping of specimens in the morphometric and enviromental space recovered by our mtDNA species delimitation

Supplementary Table S7a. Character loadings, eigenvalues and percentage of variance explained for principal components (PCI–II) of morphometric variables in eastern clade.

| Variables | PC1 | PC2 |
| --- | --- | --- |
| HW | -0.27145390 | -0.29986006 |
| HL | 0.19817704 | 0.22986006 |
| SL | 0.13483346 | 0.13832937 |
| ED | 0.04256796 | -0.09528512 |
| IND | -0.01713498 | -0.06320377 |
| UAL | 0.31909930 | -0.38166439 |
| HAL | 0.25777629 | -0.12664122 |
| THL | 0.68238827 | -0.08012108 |
| TAL | 0.36759369 | 0.24526361 |
| TL | 0.19734451 | 0.32210470 |
| FL | 0.24239685 | 0.16706204 |
| Proportion of variance | 0.3748 | 0.2029 |
| % of variation | 37.48 | 20.29 |
|  |  |  |  |

Supplementary Table S7a. Character loadings, eigenvalues and percentage of variance explained for principal components (PCI–II) of morphometric variables in western clade.

| Variables | PC1 | PC2 |
| --- | --- | --- |
| HW | -0.06979093 | 0.07973594 |
| HL | -0.15922245 | 0.15887750 |
| SL | -0.07454643 | 0.14245097 |
| ED | -0.10308788 | 0.08649269 |
| IND | -0.02191212 | 0.03191286 |
| UAL | -0.26133061 | 0.38215811 |
| HAL | -0.15530546 | -0.15530546 |
| THL | -0.43784778 | -0.43784778 |
| TAL | -0.34811346 | -0.34811346 |
| TL | -0.67146886 | -0.67146886 |
| FL | -0.31134458 | -0.31134458 |
| Proportion of variance | 0.332 | 0.1917 |
| % of variation | 33.2 | 19.17 |
|  |  |  |

Supplementary Table S7c. Character loadings, eigenvalues and percentage of variance explained for principal components (PCI–II) of environmental variables in eastern clade.

| Variables | PC1 | PC2 |
| --- | --- | --- |
| Bio1 | -0.015717411 | -0.008277502 |
| Bio2 | 0.024078811 | 0.024245101 |
| Bio3 | -0.009908581 | -0.013460223 |
| Bio4 | 0.051222299 | 0.037428103 |
| Bio5 | 0.006068017 | 0.016850287 |
| Bio6 | -0.045160030 | -0.039375932 |
| Bio7 | 0.051228047 | 0.056226219 |
| Bio8 | -0.012947867 | -0.006533222 |
| Bio9 | -0.020971087 | -0.017799107 |
| bio10 | -0.015166112 | -0.009706813 |
| bio11 | -0.016572401 | -0.009763284 |
| Bio12 | -0.519230388 | 0.528628983 |
| Bio13 | -0.018142489 | 0.093529847 |
| Bio14 | -0.063174125 | -0.002788441 |
| Bio15 | 0.023648886 | -0.002213662 |
| Bio16 | -0.063592992 | 0.298395523 |
| Bio17 | -0.203355538 | 0.018610537 |
| Bio18 | -0.129583480 | 0.281608874 |
| Bio19 | -0.508683876 | -0.532791044 |
| Proportion of variance | 0.7328 | 0.1443 |
| % of variation | 73.28 | 14.43 |

Supplementary Table S7d. Character loadings, eigenvalues and percentage of variance explained for principal components (PCI–II) of environmental variables in western clade.

| Variables | PC1 | PC2 |
| --- | --- | --- |
| Bio1 | -0.0023595881 | -0.03396163 |
| Bio2 | 0.0141430849 | 0.02485335 |
| Bio3 | -0.0038647870 | -0.01327903 |
| Bio4 | 0.0731429165 | 0.11958520 |
| Bio5 | 0.0066355446 | -0.01810788 |
| Bio6 | -0.0162394850 | -0.06956953 |
| Bio7 | 0.0228750296 | 0.05146165 |
| Bio8 | -0.0002397016 | -0.01834540 |
| Bio9 | -0.0074276086 | -0.05534892 |
| bio10 | -0.0016121156 | -0.03291453 |
| bio11 | -0.0030423604 | -0.03599881 |
| Bio12 | -0.3516130409 | 0.68977906 |
| Bio13 | -0.0672441885 | 0.10654620 |
| Bio14 | -0.0823560620 | -0.13156347 |
| Bio15 | 0.0147276954 | 0.04216460 |
| Bio16 | -0.1904547880 | 0.27385865 |
| Bio17 | -0.2583045118 | -0.29918629 |
| Bio18 | -0.1320883130 | 0.45305535 |
| Bio19 | -0.3692207130 | -0.55920411 |
| Proportion of variance | 0.9537 | 0.0018 |
| % of variation | 95.37 | 0.18 |
