# Supplementary material for: A Pan-Amazonian species delimitation: high species diversity within the genus Amazophrynella (Anura: Bufonidae)
Source: PeerJ. 2018 Jul 9;6:e4941. doi: 10.7717/peerj.4941 (PMC6042491; doi:10.7717/peerj.4941)
Supplement: Supplementary file 7 [file peerj-06-4941-s008.odt]

SUPPORTING INFORMATION

A Pan-Amazonian species delimitation: integrative methods reveals high species diversity within the genus Amazophrynella (Anura: Bufonidae)

APPENDIX 8 – Discriminant function analyses in both clades (eastern and western) revealed a grouping of specimens in the morphometric and environmental space recovered by our mtDNA species delimitation

Supplementary Figure S8a. Discriminant function analyses (DFA) of morphometric and environmental variables. Morphological DFA: A) Eastern clade (80% successfully assigned to lineages), B) Western clade (68% successfully assigned to lineages). Environmental DFA: C) Eastern clade (65% successfully assigned to lineages), D) Western clade (81% successfully assigned to lineages). UCS were not included. Symbols and colors represents the clades recovered by the phylogenetic analyses (Fig 1).

Supplementary Table S8a. Loadings and percentage of variance explained for discriminant axes (FI–II) of morphometric variables in eastern and western clade

| Variables | Eastern clade | Western clade |
| --- | --- | --- |
| LD1 | LD1 | LD2 | LD2 |
| HW | -4.4096263 | -0.4861854 | -1.636646530 | -0.91106512 |
| HL | 3.4757813 | -0.5922777 | 0.087406319 | 0.21095188 |
| SL | 1.7285995 | -1.1395011 | -0.609828335 | -4.22917381 |
| ED | 0.4064477 | -1.0965289 | 0.119461208 | 1.99903439 |
| IND | -0.4199215 | -1.1693614 | -0.405777089 | 2.43045648 |
| UAL | 0.1502300 | 0.05038776 | -0.080492727 | 1.72793872 |
| HAL | 0.7017798 | 1.05336615 | 0.005388309 | 0.92494977 |
| THL | 0.4482686 | -0.3037778 | -0.048326649 | 1.19319080 |
| TAL | 0.6212419 | 0.39058056 | 0.495730810 | -1.74431165 |
| TL | -0.3558338 | -0.1715195 | 0.193640057 | -0.08836344 |
| FL | -0.3921640 | 0.25205149 | 0.387254729 | -0.54678327 |
| Proportion of variance | 0.728 | 0.5146 | 0.2650 | 0.2023 |
| % of variation | 72.8 | 51.46 | 26.50 | 20.23 |

Supplementary Table S8b. Loadings and percentage of variance explained for discriminant axes (FI–II) of environmental variables in eastern and western clade

| Variables | Eastern clade | Western clade |
| --- | --- | --- |
| LD1 | LD1 | LD2 | LD2 |  |
| Bio1 | 0.1679008634 | 0.721457017 | 0.142555986 | 0.470151461 |  |
| Bio2 | 0.0960444575 | 0.441886826 | -0.090670815 | 0.906037633 |  |
| Bio3 | 0.1112724104 | -1.28719437 | -0.541199727 | -0.780255391 |  |
| Bio4 | -0.000232325 | -0.03288606 | -0.035187178 | 0.004400453 |  |
| Bio5 | -1.024017206 | -0.34798144 | -0.242970651 | -0.047810823 |  |
| Bio6 | -0.427340431 | -0.15384556 | -0.123503769 | 0.393922911 |  |
| Bio7 | 0.1533629836 | 0.038333601 | 0.095675758 | -0.446937305 |  |
| Bio8 | -1.278565421 | -0.25480801 | -1.057366834 | 0.555689115 |  |
| Bio9 | 0.0503488467 | -0.19259567 | -0.788218804 | -0.470717437 |  |
| bio10 | 0.0503488467 | 0.152668255 | 1.014436945 | 0.051438646 |  |
| bio11 | 2.0467316649 | 0.058457915 | 0.385811847 | -0.154496167 |  |
| Bio12 | 0.0069951698 | 0.007463796 | 0.021035091 | -0.008171557 |  |
| Bio13 | -0.036628405 | -0.00551325 | -0.055886813 | -0.023283998 |  |
| Bio14 | -0.052062471 | 0.065899019 | 0.008574135 | 0.119854471 |  |
| Bio15 | 0.2353581680 | -0.00892154 | 0.049385216 | -0.461987948 |  |
| Bio16 | -0.000794821 | -0.00975413 | -0.039964703 | 0.034561855 |  |
| Bio17 | 0.0022415514 | -0.03475757 | -0.010383787 | -0.068533578 |  |
| Bio18 | 0.0073209964 | 0.004481399 | 0.005739185 | 0.019366441 |  |
| Bio19 | -0.005116643 | -0.00601574 | -0.003106700 | 0.001336635 |  |
| Proportion of variance | 0.7062 | 0.7910 | 0.1345 | 0.1269 |  |
| % of variation | 70.62 | 79.10 | 13.45 | 12.69 |  |
